# Novel Diagnostic and Predictive Biomarkers in Pancreatic Adenocarcinoma

**DOI:** 10.3390/ijms18030667

**Published:** 2017-03-20

**Authors:** John C. Chang, Madappa Kundranda

**Affiliations:** 1 Division of Radiology, Banner MD Anderson Cancer Center, Gilbert, AZ 85234, USA; 2 Division of Medical Oncology, Banner MD Anderson Cancer Center, Gilbert, AZ 85234, USA; madappa.kundranda@bannerhealth.com

**Keywords:** pancreatic ductal adenocarcinoma, biomarker, serum, imaging

## Abstract

Pancreatic ductal adenocarcinoma (PDAC) is a highly lethal disease for a multitude of reasons including very late diagnosis. This in part is due to the lack of understanding of the biological behavior of PDAC and the ineffective screening for this disease. Significant efforts have been dedicated to finding the appropriate serum and imaging biomarkers to help early detection and predict response to treatment of PDAC. Carbohydrate antigen 19-9 (CA 19-9) has been the most validated serum marker and has the highest positive predictive value as a stand-alone marker. When combined with carcinoembryonic antigen (CEA) and carbohydrate antigen 125 (CA 125), CA 19-9 can help predict the outcome of patients to surgery and chemotherapy. A slew of novel serum markers including multimarker panels as well as genetic and epigenetic materials have potential for early detection of pancreatic cancer, although these remain to be validated in larger trials. Imaging studies may not correlate with elevated serum markers. Critical features for determining PDAC include the presence of a mass, dilated pancreatic duct, and a duct cut-off sign. Features that are indicative of early metastasis includes neurovascular bundle involvement, duodenal invasion, and greater post contrast enhancement. 18-F-fluorodeoxyglucose (18-FDG) radiotracer uptake and changes following treatment may predict patient overall survival following treatment. Similarly, pretreatment apparent diffusion coefficient (ADC) values may predict prognosis with lower ADC lesions having worse outcome. Although these markers have provided significant improvement in the care of pancreatic cancer patients, further advancements can be made with perhaps better combination of markers or discovery of unique marker(s) to pancreatic cancer.

## 1. Introduction

Pancreatic ductal adenocarcinoma (PDAC) affects 53,000 new patients each year in the USA, and is the fourth leading cause of cancer death in the USA. Of the new cases each year, almost all of them are expected to die from the disease [1]. Complete resection is the only treatment considered as potentially curative, but only 15–20 percent of patients are candidates for resection at diagnosis [2]. Even with complete resection in early stage disease, five-year survival is only 25–30 percent for node-negative, and 10 percent for node-positive disease [3].

The poor prognosis of PDAC is a result of its complex biology. Although Kirsten ras oncogen (KRAS) mutations have been identified in nearly all PDAC and precursor lesions—pancreatic intraepithelial neoplasia (PanIN), intraductal papillary mucinous neoplasm (IPMN), and mucinous cystic neoplasm (MCN)—the additional involved signaling pathways make its behavior difficult to predict [2,4,5,6]. When compared to other cancers like colorectal cancer which can harbor a KRAS mutation; the growth, response rates and prognosis of PDAC are significantly worse. In colorectal cancer, the five-year survival based on stage at diagnosis ranges from 5.7% to 74% [7]. In contrast, the five-year survival of treated PDAC ranges from 2.8% to 31.4% for resectable patients, and 0.6% to 3.8% for nonresectable patients [8]. This differences underlines our lack of clear understanding of PDAC biology.

One potential modality to improve outcomes is through cancer screening. Currently malignancies with screening include colonoscopy for CRC, mammography for breast cancer, and prostate-specific antigen (PSA) for prostate cancer. Other prognostic and predictive markers include estrogen receptor (ER)/progesterone receptor (PR)/human epidermal growth factor receptor 2 (HER2) for breast cancer, KRAS mutation for colon cancer, and anaplastic lymphoma kinase (ALK) mutation for lung cancer. However, no such effective screening technique or biomarker has been identified for PDAC at this time [9]. The goal of this review is to evaluate and understand current and emerging biochemical and imaging techniques that can provide important screening and predictive functions in the care of pancreatic cancer patients.

## 2. Traditional Serum Biomarkers

### 2.1. CA 19-9 for Detecting Pancreatic Cancer

The most commonly used and most extensively validated serum biomarker for detecting pancreatic cancer is carbohydrate antigen 19-9 (CA 19-9). Carbohydrate antigen 19-9 is a sialylated Lewis blood group antigen that is absent from the blood stream of 5%–10% of the population who are unable to express sialylated Lewis antigens [10]. Although CA 19-9 is the most commonly used antigen for detecting pancreatic cancer, it is also elevated in a variety of other conditions including malignancies such as cholangiocarcinoma, hepatocellular carcinoma, and colorectal adenocarcinoma as well as nonmalignant processes such as pancreatitis, pseudocyst, choledocholithiasis, and cirrhosis [11]. Because of the rise in CA 19-9 from these other conditions, the sensitivity and specificity for detecting pancreatic cancer in symptomatic patients ranges from 79% to 81% and 82% to 90% respectively [11]. The positive predictive value (PPV) and negative predictive value (NPV) for PDAC in symptomatic patients were reported to be 72% and 81%–96%, respectively [12,13] (Table 1). Another confounding factor to using CA 19-9 as the sole determinant is the fact that multiple commercial kits are available without a standard, resulting in additional variation [14]. This has limited the application of CA 19-9 as a stand-alone test for diagnosis. For patients with elevated CA 19-9, the positive result needs confirmation with an alternative test such as endoscopic ultrasound (EUS) or diagnostic imaging (typically computed tomography, CT) [15].

Studies have also evaluated the applicability of CA 19-9 for screening populations for pancreatic cancer, for determining resectability, and for predicting prognosis. In several large studies, CA 19-9 has been measured in asymptomatic patient population to screen for pancreatic cancer. Unfortunately, due to the low prevalence of disease, the results were less than encouraging. In a large screening study of asymptomatic patients, Kim et al. screened 70,940 patients and identified 1036 patients with elevated CA 19-9 above the upper normal cut-off of 37 U/mL. Of these, only four patients had pancreatic cancer. Although the sensitivity in this study for detection was 100% with a specificity of 98.5%, the PPV was only 0.9% [16]. In a separate study of asymptomatic patients in Japan, 13,000 patients yielded only 4 pancreatic cancer (PPV 0.03%) using CA 19-9 as a marker [17] (Table 1). These reports show the limitation of using CA 19-9 to screen for pancreatic cancer due to the low incidence. However, CA 19-9 does have use in determining resectability and prognosis of pancreatic cancer patients.

### 2.2. CA 19-9 for Determining Patient Prognosis

Multiple studies have described the correlation between CA 19-9 and pancreatic cancer resectability. The comprehensive analysis of these studies was published by Ballehanina and Chamberlain [11]. Although these studies showed that CA 19-9 can differentiate resectable and nonresectable pancreatic cancer, there is variation between the thresholds identified in each study [11]. In this analysis, a cut-off threshold at 100 U/mL resulted in 60%–80% PPV for resectability (<100 U/mL) and 88%–91% PPV for unresectability (>100 U/mL). At this cut-off, there is still a high likelihood that a patient may harbor unresectable cancer despite having CA 19-9 level less than 100 U/mL.

There has been extensive study on the correlation between CA 19-9 levels with patient prognosis. Again, this has been reviewed and analyzed by Ballehanina in 2012. The threshold used by different groups varied significantly, but the grouped analysis showed that for level <37 U/mL, the median survival ranged from 22 to 40 months while patients with level >37 U/mL had median survival between 7 and 30 months [11]. More recently, studies have shown that rate of change of CA 19-9 in the preoperative setting can predict patient survival with radiographically resectable tumor masses [18]. When the absolute and the rate of change between the two measurements taken approximately 28 days apart is <50 U/mL and <1 U/mL/day, the survival advantage can be seen up to 26 months after surgery [18]. In a separate large study of the National Cancer Database, Bergquist et al. found that CA 19-9 elevation decreased overall survival of pancreatic cancer patients regardless of stage [14]. However, in early stage disease, neoadjuvant therapy followed by curative surgery eliminated the survival difference [14]. Further refinement of the prognostication was achieved when carcinoembryonic antigen (CEA) level was assessed along with CA 19-9 [19] (Table 2). Distler et al. found that patient survival depended on the elevation of CEA and CA 19-9 with the best survival seen in patients with normal levels of both markers and the worst survival in those with elevation of both CEA and CA 19-9 [19]. Data from Chinese Society of Clinical Oncology and from Japan also supported the abbreviated survival for patients with elevated CEA and CA 19-9 [20,21]. Elevation of other serum proteins such as lactate dehydrogenase (LDH), C-reactive protein (CRP), and interleukin 6 (IL-6) also portends worse outcome of pancreatic cancer patients [22,23,24]. 

## 3. Novel Serum Biomarkers

In addition to the previously mentioned serum markers, novel markers including those obtained from the blood or tumor tissue are being evaluated to provide either earlier or more accurate detection and prediction. Techniques evaluating serum genetic material detect epigenetic changes including aberrant methylation of CpG islands in DNA affecting gene expression without affecting DNA sequence, changes in microRNA (miRNA) expression profiles and various modifications of histones (Table 3). Epigenetic changes take place at the earliest stages of tumorogenesis and therefore offer new approaches for detecting and diagnosing disease [25]. Currently, there is no theory that unifies all epigenetic pathways and hence there are several ways of monitoring and detecting epigenetic changes in PDAC. Multimarker panels identify combinations of proteins to improve detection and prediction (Table 4).

### 3.1. DNA Methylation: Detection and Prognosticating

Most of the DNA methylation in the human genome occurs on the cytosine in the CpG dinucleotides. These high density CpG sequences, are often found in promoter regions of many genes, and the methylation status of these regions is governed by DNA methyltransferases (DNMTs). While hypermethylation of the promoter is associated with gene silencing; hypomethylation results in the upregulation of the corresponding gene product.

Multiple studies have been reported regarding the potential of DNA methylation for detecting pancreatic cancer. Pedersen et al. used a two-step process to evaluate the peripheral blood leukocyte DNA from 132 PDAC patients and 60 healthy controls. The initial step identified a panel of 5-CpG sites—interleukin 10 (IL-10_P348), lipocalin 2 (LCN2_P86), zeta-chain associated kinase (ZAP70_P220), absent in melanoma 2 (AIM2_P624) and T-cell acute lymphoblastic leukemia 1 (TAL1_P817)—which was then tested in a validation set to yield sensitivity and specificity of 72% and 70%, respectively [26]. Study by Dauksa et al. evaluated the whole blood DNA from 30 PDAC patients and 49 matched controls for CpG sites in the promoters of tumor suppressor genes *p16*, retinoic acid receptor β (*RARβ*), tumor necrosis factor (TNF) receptor superfamily member 10C (*TNFRSF10C*), adenomatous polyposis coli (*APC*), apoptotic chromatin condensation inducer 1 (*ACIN1*), death-associated protein kinase 1 (*DAPK1*), heparan sulfate glucosamine 3-*O*-sulfotransferase 2 (*3OST2*), B-cell lymphoma 2 (*BCL2*) and *CD44* [27]. They also examined the methylation status of long interspersed element-1 (LINE-1) and Arthrobacter luteus (Alu) repeats. The methylation levels of *TNFRSCF10C* and *ACIN1* correlated with poor patient survival, while methylation of LINE-1 and Alu repeats were decreased in PDAC patients relative to healthy controls. Other genes have been evaluated with promising initial results, although the sensitivity and specificity of these serum markers remain to be validated [35].

### 3.2. Cell-Free Nucleosomes: Detection

Nucleosomes are the repeating subunits of DNA and histone proteins that constitute human chromatin. Released, intact nucleosome in serum or plasma can potentially serve as diagnostic disease biomarker, as elevated levels of cell-free (cf) nucleosomes have been reported in various cancers including PDAC [36,37]. Serum cf nucleosome levels and epigenetic profiles differ between PDAC and the control population. This difference could potentially be used for early detection of PDAC [28]. While no single cf nucleosome biomarker outperformed CA 19-9, combining these markers with CA 19-9 can produce a highly sensitive and specific biomarker panel. Therefore, it may be reasonable to hypothesize that with a broader range of assays these epigenetic markers maybe useful in diagnosing asymptomatic disease.

### 3.3. MicroRNAs: Detection

MicroRNAs are 19–25 nucleotides long, non-coding RNAs that regulate gene expression post-transcriptionally. MicroRNA deregulation have been implicated in the oncogenesis of multiple tumors and the associated invasive, metastatic process [38]. MicroRNA regulates genetic expression by decreasing mRNAs to decrease the translation of mRNAs into effector proteins. As miRNA is transcribed from DNA, they are regulated by DNA methylation and histone acetylation. Thus, miRNA and epigenetic control form a feedback loop to maintain proper cellular signaling. Currently, the techniques of evaluating miRNA limit wide-spread clinical applicability as detection requires quantitative real-time polymerase chain reaction (RT-PCR), next-generation DNA sequencing, and other custom built platforms. These techniques have identified thousands of miRNAs whose aggregate expression pattern varied significantly. In several studies, the difference between benign and malignant pancreatic disease allowed identification of several four-sequence panels. Further validation of these panels will be needed before wide-spread clinical use [29,39].

### 3.4. Cell-Free Tumor DNA: Early Response Assessment

Cell-free nucleic acids (cf NAs) including cell free DNA (cf DNA) is another novel technique based on liquid biopsies that has been explored for pancreatic cancer. Kinugasa et al. demonstrated that the measurements of KRAS mutation in patients with pancreatic cancer appeared to be an early monitoring tool for treatment efficacy [30]. Our initial pilot study in patients with pancreatic cancer demonstrated cf DNA could detect responses reliably prior to changes seen on conventional imaging [40]. If this can be validated in an ongoing study, cf DNA holds promise for being sensitive, specific, and non-invasive tool for clinical decision making and clinical investigations. 

### 3.5. Multimarker Panels for Detection

Due to the uniform poor outcome in pancreatic cancer patients, extensive research has been dedicated to identifying better serum biomarkers. Research has shown that sensitivity and specificity of multimarker panel are better than that of CA 19-9 alone. However, these panels have only been evaluated in single institutions and require much more extensive validation across different institutions.

In general, these panels search through different categories of proteins, signaling molecules, and enzymes. Brand et al. searched through a panel of 78 proteins to generate a limited panel of markers to identify pancreatic cancer patients [31]. They found that CA 19-9, intercellular adhesion molecule 1 (ICAM-1), and osteoprotegerin (OPG) are selective for pancreatic cancer, but not lung, breast, or colon. In a validation set consisting of pancreatic cancer patients, patients with benign pancreatic disease, and healthy patients, the panel had sensitivity and specificity of 78% and 94%, respectively for pancreatic cancer [31]. In a separate study, Chang et al. identified CA 19-9, osteopontin (OPN), chitinase 3-like 1 (CHI3L1) as the marker panel that resulted in significant improvement in sensitivity in detecting pancreatic cancer from a cohort of stage II/III patients [32].

Although these marker panels improve the detection of pancreatic cancer, they may not be applicable for actual screening or prediagnostic assessment for early detection [33]. The study from Lokshin group evaluated the feasibility of their marker panel to detect pancreatic cancer before diagnosis using the prostate, lung, colon, and ovarian cancer screening cohort, but found that the markers performed worse than CA 19-9 in the prediagnostic setting. Instead, they re-examined their panel and found that CA 19-9, CEA, and fragments of cytokeratin 21 (Cyfra 21-1) outperformed CA 19-9 alone in the prediagnostic setting. When evaluating patients at less than 1 year before diagnosis, CA 125 also showed potential. This data shows that as pancreatic cancer evolves, either the neoplastic cells or the cells in the microenvironment evolve in their protein/marker expression.

The meta-analysis by Zhang et al. evaluated the influence of the combination of markers, the thresholds of markers, and the techniques applied for detection on the sensitivity and specificity of detection [41]. Decreasing the CA 19-9 threshold from 37 to 35 U/mL resulted in slightly lowering the sensitivity and increasing specificity, an unexpected result perhaps due to the small change in threshold and the moderate heterogeneity of the analyzed reports [42]. In terms of detection technique, enzyme-linked immunosorbent assay (ELISA) is slightly more sensitive but less specific at detecting CA 19-9 than chemiluminescence immunoassay, but similar for carbohydrate antigen 242 (CA 242) and CEA [41]. These sensitivity and specificity may be related to the detecting antibody that was employed. When these tests are combined, the combination of CA 19-9 and CA 242 yielded the highest sensitivity without sacrificing the specificity (89% sensitivity; 75% specificity); the highest specificity (0.93) resulted when all three markers (CA 19-9, CEA, CA 242) are elevated at the cost of lowering the sensitivity (0.5) [41].

A separate report by Gu et al. also studied multimarker panel consisting of CA 19-9, CA 242, CA 125, and CEA [34]. Individually, CA 19-9 had the highest sensitivity (82.7%), but CA 242 had the highest specificity (90%). When the four markers were combined, the final sensitivity rose to 90.4% while specificity rose to 93.8%. However, what was unclear was the method of combining these four markers; no specific formula was mentioned within the article. The study also showed elevation of these markers above the cut-off resulted in shorter survival for the patients treated with chemoradiation [34].

Differentiating pancreatic cancer from inflammatory masses can be extremely difficult given the similarities of their imaging appearance. The study by Chang et al. suggests that co-elevation of immunoglobulin G4 (IgG4) (≥280 mg/dL) with CA 19-9 (≤85 U/mL) yielded the best accuracy for detecting autoimmune pancreatitis related mass [43]. Despite the inflammatory changes associated with chronic pancreatitis, the inflammatory markers CRP and IL-6 remain lower than stage II–IV pancreatic cancer patients [24].

### 3.6. Multimarker Panels: Prognostication

In terms of prognostication, the multimarker panels also show improved triaging of patients. In stage I and II pancreatic cancer patients, those who have elevated CA 19-9 postoperatively demonstrated decreased overall survival compared to those without CA 19-9 elevation [44]. In patients with elevated CA 19-9 postoperatively, the survival is further differentiated by the elevation of CEA with the shortest overall survival seen in those with elevation of both CA 19-9 and CEA [44]. This combination was also applied to pretherapeutic setting to assess overall cancer-related survival. The linear sum of these two markers better differentiated patient survival than the product of these two markers or the individual markers alone [45]. For patients with CEA ≥1000 U/mL, the co-elevation of CEA and CA 125 preoperatively predicted shorter post-operative overall survival than those without [46]. In stage II/III patients, elevated CEA and CA 125 resulted in shorter survival by 4 months [32]. The poor outcome with elevated CEA was also seen in stage III/IV patients [47].

## 4. Imaging Biomarkers

Imaging technology has made significant progress over the past two decades and has resulted in images acquired at higher spatial and temporal resolution. This provides additional dimensions of tumor biology that has only recently been utilized for detection and prognostication. The focus of this section will be on reviewing the recent reported findings of pancreatic cancer and for predicting survival and response of pancreatic cancer patients.

### 4.1. Imaging Markers for Detecting Pancreatic Cancer

Imaging studies for assessing pancreatic cancer are usually obtained for either direct suspicion for pancreatic cancer, nonspecific abdominal pain, pancreatitis, or follow up of pancreatic abnormalities [48]. On reviews of prediagnostic CT images of pancreatic cancer patients and control studies, there are multiple features that raise suspicion for pancreatic cancer, many of which also overlap with pancreatitis [48,49]. These features include a hypoattenuating mass, duct dilatation, duct cut-off, and upstream pancreatic atrophy [48,49]. The sensitivity and specificity of each feature is listed in Table 5 [49]. However, these features are likely to be overestimates given that the study contained 20 pancreatic cancer cases, 12 chronic pancreatitis cases, and 38 normal cases [49]. In the general population and given the low prevalence of pancreatic cancer, the sensitivities and specificities are likely to vary from the reported values.

With regard to pancreatic masses, endoscopic ultrasound-guided fine needle aspiration (EUS/FNA) analysis of these masses have shown that 74% of masses in patients with obstructive jaundice was due to malignancy, but this rate drops to 50% for patients showing only a pancreatic mass without obstructive jaundice [50]. For non-jaundiced patients with smaller masses that are ≤2.5 cm in size, the overall rate of malignancy is only 32%, and this rate drops to 16% for masses ≤1.5 cm in size [51]. In patients with chronic pancreatitis, the incidence further drops to 9.5% for patients with obstructive jaundice and 4% for non-obstructed patients with a mass alone as opposed to 39% and 22% respectively for patients without chronic pancreatitis [50].

### 4.2. Computed Tomography Imaging Markers for Predicting Patient Outcome

**Figure 1:**
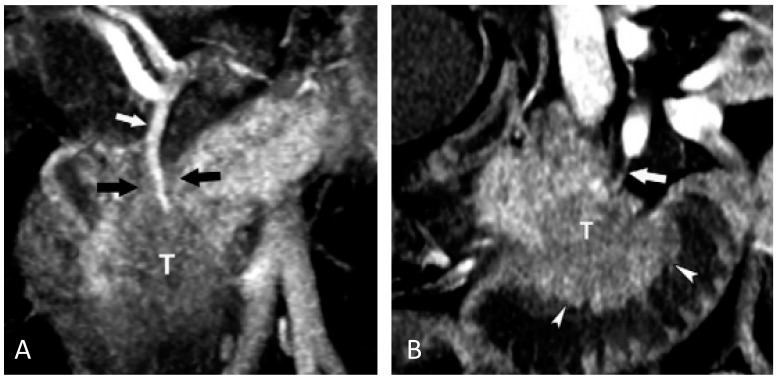
Examples of extrapancreatic perineural invasion and duodenal invation. (**A**) Pancreatic head mass (T) encasing the gastroduodenal artery (GDA) (white arrow) show as the perivascular tissue (black arrow) along the anterior nerve plexus. (**B**) Pancreatic head mass (T) invading the duodenal wall (arrowhead), but sparing the posterior-inferior pancreaticoduodenal artery (arrow). Reproduced with permission from [53].

Recent imaging research has begun to discover imaging features that may predict patient response to surgery and chemotherapy. Due to the significant morbidity and the poor overall long-term survival of pancreaticoduodenectomy, it is critical to appropriately triage even early stage patients [52]. In patients who are resectable by known CT criteria, the overall progression free survival decreases dramatically with the presence of perineural or duodenal invasion (median overall survival without either finding: 237 days; with either finding: 58 days) [53]. Examples of these from Chang et al. [53] are shown in Figure 1. In addition to early invasion of nerves and vessels, delivery of the chemotherapy to the cells are also important.

Chemotherapy accessibility depends on the perfusion of the tumor and the available uptake receptors, which ultimately influence patient response to chemotherapy (Table 6). From the study of Koay et al. using intraoperative gemcitabine infusion as a model, the important factors of response to therapy related to the expression of human equilibrative nucleotide transporter (hENT) and the diffusive transport as measured by the normalized area under curve of preoperative pancreatic CT scan [54]. These findings suggest that uptake of drug into the cells and the delivery of the drug to the extracellular environment are important factors to patient response. Chemotherapy perfusion can be inferred by the level of enhancement of the tumor mass after contrast administration. The enhancement of the pancreatic masses is directly correlated with tumor expression of vascular endothelial growth factor (VEGF) and microvessel density (MVD), but inversely correlated with fibrosis [55]. In a separate report by Wang et al., as PDAC increases in grade, the enhancement intensity decreases relative to the adjacent parenchyma; the tumor also shows increasing MVD and cystic areas [56]. These imaging changes can ultimately be used to predict the response of patients to chemotherapy. Fukukura et al. identified stronger post contrast enhancement as a marker that indicated better survival after treatment [57]. The survival advantage of patients with greater enhancement in the three phases of contrast administration is three to four times that of the patients with lower enhancement [57]. Kim et al. also found that masses (either in the pancreas or metastatic in the liver) with stronger post contrast enhancement had better response to various administered chemotherapy [58]. For pancreatic masses, 31.5 Hounsfield unit (HU) enhancement during the arterial phase of contrast enhancement yielded sensitivity of 62.8% and specificity of 91.3% for response rate [58]. For liver metastasis, 18 HU enhancement during the arterial phase yielded sensitivity of 76% and specificity of 85.7% for response rate [58]. It is interesting that 11 of the 101 patients showed discordant enhancement pattern between the pancreatic primary and liver metastasis, corresponding to potential heterogeneity of the tumor cells. In a separate study of 79 patients who had received curative resection of pancreatic tumor, the higher pretreatment enhancement of the tumor mass relative to the enhancement of adjacent pancreatic parenchyma resulted in longer overall survival (>0.9 of the parenchymal enhancement; 28.5 vs. 20.3 months) [59]. These imaging findings show the potential of imaging in predicting patient response to therapy. Specifically, lower enhancement in the pretreatment setting is associated with more aggressive tumor that has shorter survival compared to those with greater enhancement.

### 4.3. Positron Emission Tomography Marker for Predicting Patient Response 

18-F-fluorodeoxyglucose (18-FDG) positron emission tomography (PET) relies on tumor expression of carbohydrate metabolic enzymes including glucose uptake transporter for the cellular uptake and processing of FDG. The total metabolic tumor volume (MTV) is a direct consequence of the alterations in the expression levels of glucose transporter 1 (GLUT-1), fructose bisphosphate aldolase A (ALDOA), and fructose bisphosphatase 1 (FBP1) [60]. In PDAC, the level of gene expression of GLUT-1 (the most common glucose transporter expressed in malignancy) does not correlate with maximum standardized uptake value (SUVmax) or the grade of tumor [61]. However, the level of expression before adjuvant therapy is inversely associated with the outcome for stage I and II patients who had undergone curative resection followed by adjuvant chemoradiation therapy [61]. This relationship is also true in patients with downstaged, borderline resectable and locally advanced cancers [62]. The lack of direct correlation between GLUT-1 gene expression and SUVmax is intriguing in that SUVmax may also be influenced by the downstream molecules and not simply by the expression of GLUT-1. 

Despite the lack of direct correlation, preoperative SUVmax is a predictive marker for PDAC patient survival (Table 7). In stage I and II patients, lower 18-FDG uptake (<5) is associated with longer overall survival (28 months) than those with high 18-FDG uptake (16 months) [63]. In the same study, the authors found that 18-FDG uptake is correlated with higher grade lesions [63]. In a separate study of 69 non-metastatic, unresectable patients, patients with pretreatment SUVmax of greater than 5.5 resulted in significantly shorter overall survival (16.6 vs. 12.6 months) [64]. In locally advanced pancreatic cancer, patients who showed greater than 50% reduction of 18-FDG avidity achieved longer survival and better complete resection rate [65,66,67].

### 4.4. Functional Magnetic Resonance Parameters as Predictive Markers

Magnetic resonance imaging (MRI) is not typically used to assess PDAC due to the need for breath holding for acquiring good images. However, given the ability to acquire functional parameters for PDAC and the improvement in hardware, reports of functional imaging for PDAC have recently increased. The most common functional parameter to acquire is the diffusion weighted imaging which produces the apparent diffusion coefficient (ADC). In the report by Niwa et al., lower ADC was associated with shorter progression free survival, although a definite threshold was not given [68]. In a preclinical study, dynamic contrast enhanced (DCE) parameters volume transfer coefficient (Ktrans), and flux rate constant (Kep) decreased at 3 days after abraxane therapy [69]. This decrease was also accompanied by significant decrease in Ki67 protein which eventually recovered 7 days after chemotherapy [69]. These magnetic resonance (MR) findings are early, pilot level studies which will require larger patient studies for validation.

## 5. Conclusions

Pancreatic adenocarcinoma is a deadly disease with only a handful of patients who can be considered cured. At present, our lack of understanding of the biology of this disease has prevented the development truly effective therapies and clinically useful markers for screening the disease. However, as our understanding of this disease improves through future research, we can expect better markers and/or panels of markers to improve detection such that screening becomes the norm and that imaging can guide therapy by revealing the tumor microenvironment and the class of driver mutations.

## Figures and Tables

**Table 1:** Summary of pancreatic cancer detection using carbohydrate antigen 19-9 (CA 19-9) (>37 U/mL) [11,16,17].

Patient Groups	Sensitivity (%)	Specificity (%)	PPV	NPV
Symptomatic	79–81	82–90	72	81–96
Asymptomatic	100	98.5	0.03–0.9	-

NPV: negative predictive value; PPV: positive predictive value.

**Table 2:** Mean survival of patients based on preoperative elevation of CA 19-9 and carcinoembryonic antigen (CEA) [19].

CA 19-9; CEA	Mean Survival (months)
≤75 U/mL; ≤3 ng/mL	33.3
>75 U/mL; ≤3 ng/mL	28.5
or	
≤75 U/mL; >3 ng/mL	
>75 U/mL; >3 ng/mL	23.9

**Table 3:** Summary of novel serum markers.

References	Marker Class	Markers	Comments
[26]	DNA Methylation	IL-10_ P348, LCN2_P86, ZAP70_P220, AIM2_P624 and TAL1_P817	Sen: 72%; Spec: 70% for detecting PDAC. Never-smoked population
[27]	DNA Methylation	TNFRSCF10C, ACIN1	Hypermethylation indicates shorter survival
	DNA Methylation	Line-1 and ALU repeats	PDAC patients have decreased methylation in ALU and Line-1 CpG repeats
[28]	Cell Free Nucleosomes	5MC, H2AZ, H2A1.1, H3K4Me2, CA 19-9	AUC: 0.98; Sen: 92%; Spec: 90% for detecting PDAC
[29]	MicroRNA	miR-223	Elevated miR-223 increased risk for PDAC
[30]	Cell Free DNA	KRAS mutation	77% concordant between actual biopsy and liquid biopsy in detecting mutation

Sen: Sensitivity; Spec: Specificity; AUC: Area under curve; PDAC: Pancreatic ductal adenocarcinoma.; IL-10: Interleukin 10; LCN2: Lipocalin 2; ZAP70: Zeta-chain-associated protein kinase 70; AIM2: Absent in melanoma 2; TAL1: T-cell acute lymphoblastic leukemia 1; TNFRSCF10C: Tumor necrosis factor (TNF) receptor superfamily member 10C; ACIN1: Apoptotic chromatin condensation inducer 1; Line-1: Long interspersed element-1; ALU: Arthrobacter luteus; 5MC: 5-methylcytosine; H2AZ: Histone 2A.Z; H2A1.1: Histone macro 2A1.1; H3K4Me2: Histone H3 dimethyl Lys4 ; miR: MicroRNA; KRAS: Kirsten ras oncogen.

**Table 4:** Summary of multimarker panels.

References	Markers (Protein)	Comments
[31]	CA 19-9, ICAM-1, OPG	Sen: 78% and Spec: 94% in detecting pancreatic cancer
[32]	CA 19-9, OPN, CHI3L1	Sen: 93% in detecting pancreatic cancer Studied in stage II/III patients
[33]	CA 19-9, CEA, Cyfra 21-1	Increased sensitivity of detection at high level of specificity in asymptomatic subjects Studied in prostate, lung, colorectal, and ovarian screening study population
[34]	CA 19-1, CA 242, CA 125, CEA	Sen: 90% and Spec: 94% Studied in patients undergoing chemoradiation

Sen: Sensitivity; Spec: Specificity; ICAM-1—Intercellular adhesion molecule 1; OPG: Osteoprotegerin; OPN: Osteopontin; CHI3L1: Chitinase 3-like 1; Cyfra 21-1: Fragments of cytokerintin 21; CA 19-1: Carbohydrate antigen 19-1; CA 242: Carbohydrate antigen 242.

**Table 5:** Computed tomography (CT) features predicting the presence of pancreatic cancer [49].

Imaging Finding	Sensitivity (%)	Specificity (%)	Accuracy
Focal mass	75	84	0.81
Pancreatic duct dilation	50	78	0.7
Duct interruption	45	82	0.71
Upstream atrophy	45	96	0.81
Contour abnormality	15	92	0.7
CBD dilation	5	92	0.67

CBD: Common bile duct.

**Table 6:** Postcontrast enhancement intensity and patient survival.

Reference	Patient Population	Contrast Phase: ∆HU from Unenhanced CT	Survival
[57]	Unresectable	Arterial: ≥28	20.8 vs. 10.9 months
	Unresectable	Portovenous: ≥34	20.8 vs. 10.9 months
	Unresectable	Delayed: ≥36	20.8 vs. 11.8 months
[57]	Resectable	Arterial: ≥48	60.8 vs. 18.3 months
	Resectable	Portovenous: ≥56	60.8 vs. 18.3 months
	Resectable	Delayed: ≥57	60.8 vs. 16.4 months
[58]	Unresectable (Pancreatic mass enhancement)	Arterial: ≥31.5	Sen: 62.8%; Spec: 91.3% for predicting response to chemotherapy
	Unresectable (Liver mass enhancement)	Arterial: ≥18	Sen: 76%; Spec: 85.7% for predicting response to chemotherapy

∆HU: Changes in Hounsfield unit.

**Table 7:** Maximum standardized uptake value (SUVmax) and changes in SUVmax correlates with patient therapeutic outcome.

Reference	Patient Population/Therapy	SUV Threshold	Overall Survival
[63]	Stage I/II:Curative Resection	<5	28 vs. 16 months
[64]	Locally Advanced/Chemoradiation	≤5.5	16.6 vs. 12.6 months
[66]	Locally Advanced/Chemoradiation	≥50% decrease following therapy	1 year survival of 87% vs. 28%
